# Impact of the COVID-19 Pandemic on Older People's Loneliness: Findings from a Longitudinal Study between 2019 and 2021 among Older Home-Dwellers in Finland

**DOI:** 10.1007/s12603-023-1949-2

**Published:** 2023-08-01

**Authors:** M.T. Knuutila, L. Rautiainen, T.E. Lehti, H. Karppinen, H. Kautiainen, T.E. Strandberg, H. Öhman, N.M. Savikko, A.H. Jansson, K.H. Pitkälä

**Affiliations:** 1 Social Services and Health Care, City of Helsinki, Helsinki, Finland; 2 Department of General Practice and Primary Health Care, University of Helsinki, Helsinki, Finland; 3 Unit of Primary Health Care, Helsinki University Hospital, Helsinki, Finland; 4 Geriatric Medicine, University of Helsinki and Helsinki University Hospital, Helsinki, Finland; 5 City of Espoo, Elderly Care, Espoo, Finland; 6 The Finnish Association for the Welfare of Older Adults, Helsinki, Finland; 7 Tammisalontie 20 as 4, 00830, Helsinki, Finland

**Keywords:** Loneliness, COVID-19, well-being, longitudinal, older adults

## Abstract

**Objectives:**

To investigate the change in feelings of loneliness among Finnish community-dwelling older people from before the COVID-19 pandemic in 2019 to during the pandemic in 2021. Moreover, we explore the changes in other dimensions of psychological well-being (PWB) during the study period.

**Design:**

Questionnaires were mailed in the 2019 Helsinki Aging Study, a repeated cohort study. A follow-up interview was carried on over the telephone during the year 2021.

**Setting and Participants:**

A random sample of 2,917 home-dwelling older people aged 75–104 years residing in Helsinki, Finland were mailed the questionnaire. Altogether 898 participated in the follow-up.

**Measurements:**

Loneliness was measured using a single item question “Do you suffer from loneliness?”. Other items of psychological well-being were measured: ”Are you satisfied with your life?“ (yes/no), “Do you feel useful?“ (yes/no), ”Do you have a zest for life?“ (yes/no),”Do you have plans for the future?“ (yes/no), and ”Do you feel depressed?”(“rarely or never”/”sometimes”/”often or always”).

**Results:**

Altogether 898 people participated both in 2019 and 2021. The subjects' mean age was 83 years and 66% were women. Between 2019 and 2021, the prevalence of experienced loneliness increased among older home-dwellers from 26% to 30%. During two years of the pandemic feelings of loneliness (RR 1.79, 95% CI: 1.30 to 2.46) and depression (RR 1.37, 95% CI: 1.12 to 1.67) increased even adjusted with various confounders.

**Conclusion:**

Considering the impact loneliness has on health and well-being, the finding of increased feelings of loneliness among older people is alarming. Actions to combat loneliness need to be taken.

## Introduction

Older adults' loneliness is a timely topic due to the quarantining of older people during the COVID-19 pandemic. As loneliness is an independent risk factor for poor health, cognitive decline, decreased well-being, and even increased mortality (1, 2, 3, 4), the topic is acutely worthy of interest. Loneliness is defined as a subjective, negative experience, deriving from unmet expectations of quality or quantity of social relationships (5, 6). The subjective nature of the experience is essential in distinguishing loneliness from social isolation, which in turn refers to an objectively measured lack of social contact or limited social network (7).

Due to various life changes attributed to old age, such as widowhood, death of peers and weakened health, older people are especially vulnerable to loneliness (8). In Finland, a good third of older adults suffer from loneliness at least sometimes (9, 10), and, for instance, 25% to 29% of older Americans have been categorized as lonely in previous studies (4). Even though loneliness among older people is pervasive, the prevalence has been suggested to remain stable (11) or even decrease over past decades (12).

COVID-19 emerged in late 2019, and in March 2020, the World Health Organization (WHO) declared it a pandemic (13). During the first waves of the pandemic social distancing, lockdowns, and stay-at-home orders were essential in preventing the virus from spreading uncontrollably. The distancing measures were even more stringent among older people due to their heightened risk of morbidity and mortality from the virus (14). The restrictions were hypothesized to increase social isolation and further exacerbate loneliness among older people, particularly among those living alone and having functional limitations.

Psychological well-being (PWB) has been defined in WHOQOL-BREF as including dimensions such as life satisfaction, meaningful life, absence of negative feelings like depression and anxiety, and satisfaction with social relationships (15). Many stressors that emerged during the pandemic, such as fear of getting infected and dying from the virus, and bereavement of loved ones might interact with the dimensions of PWB (16).

Longitudinal studies exploring the change in older adults' loneliness and PWB are scarce and only few longitudinal studies compare data from before COVID-19 and beyond the initial months of the pandemic (17, 18). The severity of the pandemic and strictness of restriction measures have varied considerably in different countries and the need for specific country-based data has been recognized (19, 20). In the beginning of the pandemic Finnish older people were requested to avoid close contacts and going outdoors (21). Most older people followed the recommendations carefully, leading to community-dwelling older people being isolated in their own homes (22).

Our aim is to determine, in a longitudinal setting, the change in experienced loneliness among Finnish community-dwelling older people residing in Helsinki from before the COVID-19 pandemic in 2019 to during the pandemic in 2021. Moreover, we aim to explore the change in other dimensions of PWB among the participants between 2019 and 2021.

## Methods

### Study design and participants

As part of the Helsinki Aging Study (ongoing from 1989), a random sample of home-dwelling older people aged 75+ were mailed a postal questionnaire in June and August of 2019. The questionnaire was sent to 600 people from each age group of 75-, 80-, 85-, and 90-year-olds and all 95-year-olds and 100-year-olds and over. The response rate in 2019 was approximately 74% (N=1,810), based on excluding the estimated number of people who had died, moved away, or been institutionalized between the latest population census and the retrieval of the sample. We aimed to include at least 800 of the original sample into the follow-up study. Of the participants of the initial survey, all those who were alive and gave their permission were contacted, and telephone interviews were conducted during the year 2021. Altogether 898 people took part in the follow-up interviews, thus responding to the items of loneliness and PWB at both time points.

The study design was approved by the Helsinki University Hospital Ethics Committee. All participants gave their informed consent to participate in the study. Data was collected using a questionnaire and analyzed anonymously.

### Measurements

#### Demographics

The respondents' age, sex and marital status (married or cohabiting/single/divorced or separated/widowed) were determined. The respondents were surveyed on their level of income (good/moderate/poor), and level of education (categorized as less than 8 years, 8–12 years, and more than 12 years of education).

#### Loneliness and psychological well-being

Loneliness and items on psychological well-being were surveyed with identical questions in 2019 and during the follow-up in 2021.

Loneliness was measured using a direct single question item: “Do you suffer from loneliness?” with response options ”always or often,” ”sometimes,” ”seldom or never.” Two categories were formed: lonely (“always or often,” “sometimes”) and not lonely (“seldom or never”).

To include other dimensions of PWB, we asked: “Are you satisfied with your life?” (yes/no), “Do you feel useful?” (yes/no), “Do you have a zest for life?” (yes/no), and “Do you have plans for the future?” (yes/no). Furthermore, we asked: “Do you feel depressed?”(“rarely or never”/“sometimes”/“often or always”), and considered those who responded “often or always” and “sometimes” as feeling depressed in our analyses.

#### Health and physical functioning

The respondents' health was assessed using the Charlson Comorbidity Index (CCI) (23), a weighted index taking into account the number and seriousness of comorbidities. CCI was calculated based on self-reported illnesses. Furthermore, self-rated health (SRH) was determined by asking: “How is your state of health?” (healthy/moderately healthy/moderately unhealthy/unhealthy). We formed two categories, good SRH (healthy or moderately healthy) and poor SRH (moderately unhealthy/unhealthy) (24, 25).

Self-rated physical functioning was assessed: “How would you rate your ability to function or your general physical condition at the moment?” (Very good -Good/Average/Poor -Very poor).

#### Sense of security, social connectedness, and perceived treatment

To ascertain the respondents' sense of security we asked: “Do you find your life to be secure or insecure at this moment?” (very secure/moderately secure/moderately insecure/very insecure/indecisive) and dichotomized the answers to secure (very and moderately secure) and insecure (very and moderately insecure), omitting those who remained indecisive.

Regarding existing social ties we asked: “Do you have living children?” (yes/no) and “Do you have friends with whom you keep in touch regularly?” (yes/no). In order to assess the respondents' digital connectedness, we asked whether the respondents used a smartphone, a computer or the Internet (yes/no).

To assess the respondents' perception of treatment of older people, both on societal level and personally, we asked: “How, in your opinion, are older people treated in Finland?” (well/moderately/poorly) and “How have you been treated as an older person?” (well/moderately/poorly).

In order to assess the differences between those participating only in the initial survey and those completing the follow-up, we compared the groups in terms of age, sex, comorbidities (CCI), functioning and loneliness.

### Statistical analysis

Data are presented as means with standard deviation (SD) or as counts (n) with percentages (%). Group differences were evaluated using unpaired Student's t-test, Mann-Whitney U test, chi-squared test, or Fisher's exact test, as appropriate. Repeated measures of loneliness and items on psychological well-being were analyzed using generalized estimating equations (GEE) models (logit link and binomial distribution) with the unstructured correlation structure (QIC criterion for model selection). Age, sex, having living children, education, marital status, functioning, having friends, and CCI were introduced into the model as covariates, as they were hypothesized to represent risk factors for increased loneliness. The permutation method (Monte Carlo p-values) was used when the theoretical distribution of the test statistics was unknown or in the case of violation of the assumptions (e.g., non-normality). Normal distributions were evaluated graphically and with the Shapiro-Wilk W test. Stata 17.0 (StataCorp LP, College Station, TX, USA) was used for the analysis.

## Results

Altogether 898 people participated both in the initial survey and in the follow-up interview. The participants' mean age was 83 years (SD 6.0) and 589 (66%) were women. Of participants, 237 (26%) felt lonely at least sometimes in 2019. Table 1 presents the baseline characteristics of both groups in 2019. The lonely respondents were more often women and older than those not lonely, and they had lower levels of education and income. There were more widows and fewer were married or cohabiting among the lonely respondents compared to those not lonely. The lonely had weaker health, which was reflected in their higher CCI and poorer SRH compared to those not lonely. Those lonely at baseline also had poorer self-rated physical functioning compared to those not lonely. The lonely were more likely to perceive poor societal or personal treatment than the not lonely, and they were also more likely to feel insecure. The lonely were less likely to have friends with whom they kept in touch regularly compared to those not lonely. There was no difference between the groups in terms of having living children. The lonely respondents were less likely to use the internet, a computer, and a smartphone than those not lonely.

**Table 1:** Baseline characteristics of the lonely and not-lonely participants in 2019

	**Not lonely in 2019 N=661**	**Lonely in 2019 N=237**	**P value**
Women, n (%)	405 (61)	184 (78)	<0.001
Age, years, n (%)			<0.001
75–79	198 (30)	37 (16)	
80–84	155 (23)	55 (23)	
85–89	170 (26)	62 (26)	
90+	138 (21)	83 (35)	
Education, n (%)			<0.001
<8 years	140 (21)	65 (28)	
8–12 years	188 (29)	88 (37)	
>12 years	331 (50)	82 (35)	
Marital status, n (%)			<0.001
married or cohabiting	332 (50)	45 (19)	
single	54 (8)	22 (9)	
divorced or separated	95 (14)	53 (22)	
widowed	179 (27)	117 (49)	
Income, n (%)			<0.001
Good	268 (41)	50 (21)	
Moderate	379 (57)	166 (70)	
Poor	14 (2)	20 (8)	
Self-rated physical functioning, n (%)			<0.001
Good	353 (54)	68 (29)	
Moderate	249 (38)	119 (51)	
Poor	50 (8)	48 (20)	
Good self-rated health, n (%)	584 (89)	171 (72)	<0.001
Charlson Comorbidity Index^1^, mean (SD)	1.5 (1.5)	1.9 (1.6)	0.006
Feels insecure, n (%)	24 (4)	30 (13)	<0.001
Perceives poor societal treatment, n (%)	120 (19)	60 (27)	0.015
Perceives poor personal treatment, n (%)	19 (3)	22 (10)	<0.001
Has friends, n (%)	611 (94)	186 (79)	<0.001
Has living children, n (%)	538 (82)	194 (82)	0.95
Uses a computer, n (%)	442 (68)	117 (50)	<0.001
Uses the internet, n (%)	389 (66)	96 (47)	<0.001
Uses a smartphone, n (%)	326 (50)	85 (37)	<0.001

1. Charlson ME, Pompei P, Ales KL, et al. A new method of classifying prognostic comorbidity in longitudinal studies: development and validation. J Chronic Dis 1987; 40: 373–83.

Those respondents participating only in the initial survey and not in the follow-up interview differed in some respect from those completing the follow-up. Those not participating in the follow-up interview were older and had poorer functioning. On the other hand, those participating in the follow-up interview were more likely to be lonely at baseline than those not participating in the follow-up. The groups did not differ in terms of gender or CCI.

**Figure 1:**
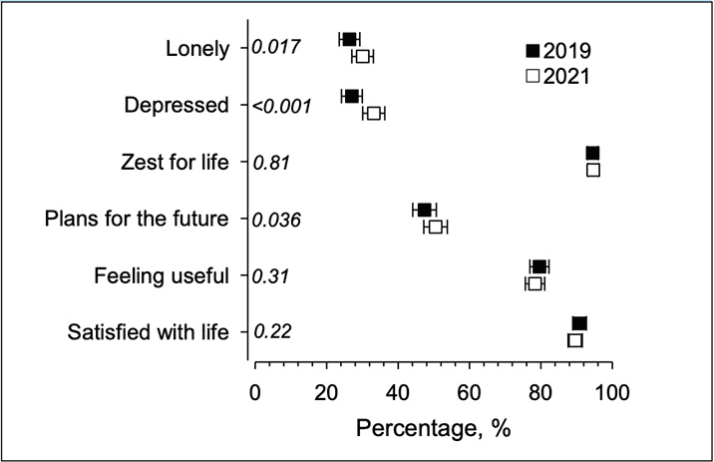
Prevalence of various dimensions of psychological well-being in 2019 (in black) and 2021 (in white) presented with 95% confidence intervals and p-values

Of participants, 26% (95% CI: 24 to 29) felt lonely before the pandemic in 2019, and 30% (95% CI: 27 to 33) felt lonely in the follow-up in 2021. Figure 1 presents the prevalence of various dimensions of PWB in 2019 and 2021. Satisfaction with life, feeling useful, having zest for life, or having plans for the future did not show any significant change during the follow-up, but the proportion feeling depressed increased between 2019 (27%; 95%CI: 24 to 30) and 2021 (33%; 95% CI: 30 to 36).

**Figure 2:**
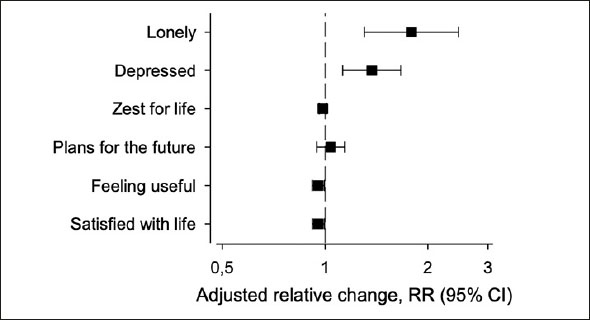
Adjusted relative change of various dimensions of psychological well-being from 2019 to 2021 presented with 95% confidence intervals

Figure 2 presents the adjusted change in various dimensions of PWB. To assess the impact of COVID-19, we calculated change in experienced loneliness and other dimensions of PWB adjusted with age, sex, having living children, education, marital status, functioning, having friends, and CCI. Between 2019 and 2021, there was a significant increase in feelings of loneliness (RR 1.79, 95% CI: 1.30 to 2.46) and feelings of depression (RR 1.37, 95% CI: 1.12 to 1.67). Additionally, there was a slight but statistically significant decrease in life satisfaction (RR 0.96, 95% CI: 0.92 to 1.00) and feeling useful (RR 0.95, 95% CI: 0.92 to 1.00). There was no statistically significant change in zest for life (RR 0.99 95% CI: 0.96 to 1.01) or having plans for the future (RR 1.04 95% CI: 0.95 to 1.04). Table 1 in supplementary materials provides information on how PWB changed according to the changes in experienced loneliness during the follow-up.

Adjusted with age, sex, having living children, education, marital status, functioning, having friends, and CCI

## Discussion

Since the onset of the COVID-19 pandemic, feelings of loneliness have increased among home-dwelling older people, from 26% feeling lonely in 2019 to 30% in 2021. Even when adjusted with various confounders, there was a significant increase in feelings of loneliness and depression, and a marginally significant decrease in life satisfaction and feeling useful among home-dwelling older people.

Results from prior studies examining the change in older adults' loneliness during COVID-19 have not been consistent, as some have suggested an increase in experienced loneliness among older people (17, 18, 26, 27), while others have suggested no change (28, 29). However, results from those very few longitudinal studies comparing pre- and peri-pandemic data on a longer time frame beyond the initial months of the pandemic are in line with ours suggesting an increase in feelings of loneliness (17, 18). Moreover, prior studies have been inconsistent concerning the increase (30) or unchanged depressive symptoms (27) during the the pandemic, but again when comparing results with those longitudinal studies conducted on a longer stretch of time, we had similar findings of increased feelings of depression (18, 31).

It is understandable that the two dimensions of PWB that changed the most were feelings of loneliness and depressive symptoms. Loneliness and depression are considered distinct but overlapping phenomena (32) and the direction of causality is suggested as being bidirectional with the two acting in a synergistic way to diminish well-being (33). One prior study (26) suggested that individuals experiencing worsened feelings of loneliness during a shelter-in-place period more likely reported worsened depressive symptoms as well. Another longitudinal study from the early stages of the pandemic suggested that experiencing loneliness was a risk for increased depressive symptoms (34).

Due to imposed restrictions and personal cautiousness, social isolation among older people undoubtedly increased dramatically during the period of COVID-19. Still, our results suggest a rather modest increase in experienced loneliness, attesting to the notion that social isolation and loneliness really are distinct concepts. A longitudinal study from the U.S. showed that older adults reported increased social isolation due to COVID-19 but had no change in loneliness (29). Furthermore, the core definition of loneliness offers one explanation to why loneliness did not increase among older people as much as we may have hypothesized. As loneliness originates from the discrepancy between desired and actual level of social contact, older people might already have set their expectations to a level where the restrictions did not have that big of an impact. Studies conducted during the pandemic including all age groups have suggested that among older people, the increase in experienced loneliness has not been as drastic, when compared to younger age groups due to greater resilience among older people (26, 35). This offers one explanation to the rather modest increase in experienced loneliness in our sample consisting only of older people.

The use of technology for social interaction may have alleviated feelings of loneliness among some respondents. As seen in our results, those digitally connected were less likely to be lonely at baseline. However, our respondents were 75+ years at baseline, and not everyone is accustomed to these means of contact. Thus, relying on technology in providing social contact during the pandemic might have deepened the existing digital divide. A study examining loneliness among older people in San Francisco during a shelter-in-place period suggested that, for many participants, technology offered a means to connect with loved ones, alleviating their loneliness (26). However, for several participants inadequate access to technology was, in fact, a reasoning they gave to their worsened loneliness (26).

It is noteworthy that our results present the net change of loneliness among the participants, i.e., some of the not-lonely respondents became lonely during the follow-up and some of the lonely became not-lonely (for details, see Table 1 in supplementary materials). Even if the positive changes balance the effect in our analyses, the increase in experienced loneliness for a significant number of participants is inarguably a public health issue (2). Loneliness has been suggested to impair health by several mechanisms: by affecting health behaviours, by causing excessive stress reactions in the body and by affecting the body's physiological repair and maintenance processes (36). Also, it has been suggested that the physiological effects of loneliness are long-lasting and might unfold over a long time (36). This in mind, even a rather modest net increase in loneliness among older people is worrying. Furthermore, even though studies have varied in terms of who were most at risk for becoming lonely during the pandemic (20), it might partly be due to underrepresentation of those in poorer health and functioning in epidemiological studies (37). Khan et al. (38) observed that older people became lonelier during the pandemic and not having a spouse, living on a lower income, and suffering from depression were risk factors for increased loneliness in that age group. Thus, another concern is that during a crisis like the COVID-19 pandemic, those affected most negatively are those already in a more vulnerable position in society. This should be taken into consideration when planning interventions.

### Strengths and limitations

The strength of our study is that it is among the few longitudinal studies investigating the impact of the pandemic on older people's loneliness and PWB by comparing pre- and peri-pandemic data within a longer time frame. Thus, our study gives an idea of the long-term consequences of the COVID-19 pandemic on older people's well-being. The response rate to the questionnaire was high and we had a good representation of the older age groups, the mean age of participants being 83 years. Furthermore, our data collection methods are more suitable for the older population, as opposed to many studies conducted during the pandemic employing web-based surveys (39).

This study also has limitations. Firstly, the finding of increased prevalence of loneliness during two years of the COVID-19 pandemic does not necessarily mean that the increase is due to the pandemic but rather the results suggest a significant association. Other participant-related factors may have had an impact on the increased loneliness. Ageing is significantly associated with increased loneliness not because of age per se but with all the negative changes that occur in late life (8). However, prior studies have suggested that the prevalence of loneliness has been stable or rather decreasing than increasing over time in older people's cohorts (11, 12) indicating that the present finding is alarming. Secondly, those participating in the follow-up assessment were a selected sample. Those in better condition had the strength to participate in the follow-up study. This, and the fact that those participating in the follow-up study were lonelier at baseline might give an underestimate of the increased loneliness during the pandemic. Furthermore, our data was collected among home-dwelling older people. Older people living in long-term care facilities have faced unique challenges during the pandemic due to, e.g., visitation bans and canceled activities (16). Therefore, our results can only truly be generalized to home-dwelling older people. Additionally, as we only had two assessments of loneliness and PWB, the results might have been influenced by the current phase of the pandemic and restrictions during the data collection time. The follow-up interviews were carried out throughout the year 2021, with more stringent restrictions during the first months of the year and then gradual relaxing of restrictions as vaccination coverage improved in Finland (40). As prior studies have suggested, loneliness was greatest during lockdown periods and decreased as soon as restrictions were relaxed (41, 42).

Our single-item direct measure poses another limitation, as it leads to question whether the results are comparable with studies employing different measures. The direct measure has been criticized for being too simplistic (32) and resulting in underreporting of loneliness due to a stigma related to loneliness (4). However, the direct measure is easy for older people (even those cognitively impaired) to understand and in our repeated cross-sectional study it has proved well-suited for older people and shown reliability and prognostic validity (24). Additionally, at baseline the data was collected by a questionnaire and follow-up interviews were carried out over the telephone, which might have influenced the responses. However, it has been argued that it is easier to admit loneliness in an anonymous questionnaire than in an interview (43), which might also give an underestimate of the increase in loneliness.

## Conclusion

We investigated the impact of the COVID-19 pandemic on older people's loneliness and psychological well-being one year into the pandemic. We witnessed an increase in feelings of both loneliness and depression since the onset of the pandemic. The observed increase in experienced loneliness among older adults is alarming given the indisputable impact loneliness has on older people's health and well-being.

## Conceptualization

Knuutila, Rautiainen, Lehti, Strandberg, Pitkälä; Methodology: Knuutila, Rautiainen, Lehti, Karppinen, Kautiainen, Strandberg, Öhman, Savikko, Jansson, Pitkälä; Formal analysis and investigation: Knuutila, Kautiainen, Pitkälä; Data curation: Knuutila, Kautiainen, Pitkälä; Writing - original draft preparation: Knuutila, Kautiainen, Pitkälä; Writing - review and editing; Knuutila, Rautiainen, Lehti, Karppinen, Kautiainen, Strandberg, Öhman, Savikko, Jansson, Pitkälä; Funding acquisition: Knuutila, Strandberg, Pitkälä; Software: Kautiainen, Pitkälä; Supervision: Pitkälä.

## Electronic Supplementary Material

Supplementary material, approximately 15.7 KB.

*Funding*: This work was supported by VTR funding (Helsinki University Hospital, Helsinki City, grant numbers N.A.), Uulo Arhio Foundation (grant number N.A.), the Päivikki and Sakari Sohlberg Foundation (grant number N.A.), and The Finnish Association for General Practice (grant number N.A.) The sponsors had no role in study design, data collection and analysis, decision to publish or preparation of the manuscript. Open Access funding provided by University of Helsinki including Helsinki University Central Hospital.

*Declaration of conflicts of interest:* The authors declare no conflicts of interest.

*Ethical approval:* The study design was approved by the Helsinki University Hospital Ethics Committee (registration number HUS/508/2019). All participants gave their informed consent to participate in the study. Data was collected using a questionnaire and analyzed anonymously.
