# Supplementary material for: Impact of the COVID-19 Pandemic on Older People's Loneliness: Findings from a Longitudinal Study between 2019 and 2021 among Older Home-Dwellers in Finland
Source: J Nutr Health Aging. 2023 Aug 1;27(8):619–25. doi: 10.1007/s12603-023-1949-2 (PMC12877599; doi:10.1007/s12603-023-1949-2)
Supplement: Supplementary file 1 — Supplementary material, approximately 15.7 KB. [file mmc1.docx]

Supplementary materials

Table 1. Stability of loneliness among participants between 2019 and 2021 and their PWB scores in 2019 and 2021.

| Loneliness 2019-2021 | Number (%) | PWB score in 2019  Mean (SD) | PWB score in 2021  Mean (SD) |
| --- | --- | --- | --- |
| Not lonely at either time points | 548 (61) | 0.89 (0.14) | 0.89 (0.14) |
| Became lonely | 113 (13) | 0.83 (0.15) | 0.69 (0.20) |
| Recovered from loneliness | 80 (9) | 0.64 (0.24) | 0.82 (0.18) |
| Lonely at both time points | 125 (17) | 0.55 (0.26) | 0.56 (0.25) |

PWB (psychological well-being) score was determined by a validated scale (1). It was calculated on the basis of six questions regarding psychological well-being: ‘Are you satisfied with your life?’ (yes/no), ‘Do you feel useful?’ (yes/no), ‘Do you have a zest for life?’ (yes/no), and ‘Do you have plans for the future?’ (yes/no), ‘Do you feel depressed?’ (rarely or never/sometimes/often or always) and ‘Do you suffer from loneliness?’(rarely or never/sometimes/often or always). For the first four questions, yes yielded 1 point and no yielded 0 points. The two latter three-step questions gave either 0 points, 0.5 points or 1 point, a more positive answer yielding a greater score. The raw points of each question were totaled, and the sum of the raw points was then divided by the number of questions answered, providing a score between 0 and 1, with a greater score indicating better PWB, as suggested in the original scale (1)

1. Routasalo PE, Tilvis RS, Kautiainen H, Pitkala KH. Effects of psychosocial group rehabilitation on social functioning, loneliness and well-being of lonely, older people: Randomized controlled trial. *J Adv Nurs* 2009; 65: 297-305. https://doi.org/10.1111/j.1365-2648.2008.04837.x
